# Evaluating bio-physicochemical properties of raw powder prepared from whole larvae containing liquid silk of the domestic silkworm

**DOI:** 10.3389/fnut.2024.1404489

**Published:** 2024-06-05

**Authors:** Shusuke Hashimoto, Maki Yamazaki, Hiroshi Uehara, Shinya Yamazaki, Masakazu Kobayashi, Takeshi Yokoyama, Kenjiro Yazawa, Kunihiro Shiomi

**Affiliations:** ^1^Faculty of Textile Science and Technology, Shinshu University, Ueda, Japan; ^2^Morus Inc., Tokyo, Japan; ^3^Department of Food Technology, Nagano Prefecture General Industrial Technology Center, Nagano, Japan; ^4^Department of United Graduate School of Agricultural Science, Tokyo University of Agriculture and Technology, Fuchu, Japan

**Keywords:** entomophagy, oral administration, fibroin, silk, *Bombyx mori*

## Abstract

The domestic silkworm, *Bombyx mori*, has been widely used in silk production for centuries. It is also used as a bioreactor by the textile and pharmaceutical industries to mass produce recombinant bioactive proteins containing silk-based materials. Furthermore, silkworms are well-known as a source of food and have also been orally administered to prevent and treat several human disorders. In this study, we aimed to investigate the inherent bio-physicochemical properties of edible silkworms to accurately evaluate their clinical and nutritional potential. We prepared raw powder from whole larvae of silkworm. The yield rate of the powder derived from dried larvae was almost 100% (98.1–99.1% in replicates). As “percentage yield” translates to “Budomari” in Japanese, this raw powder was named “B100rw.” We further prepared B100dn that was denatured through autoclaving. Thereafter, we examined whether B100rw sustained the original bio-physicochemical properties by comparing it with B100dn. There was no significant difference in nutritional content between B100rw and B100dn. B100rw contained proteins derived from silkworm larvae and mulberry leaves, whereas the proteins of B100dn were mostly degraded. On measuring the enzymatic activity of both powders using trehalase as an indicator enzyme, B100rw was found to maintain trehalase activity. B100rw also maintained a random coil conformation, similar to that of liquid silk. This suggested that B100rw sustained the unique bio-physicochemical properties of living larvae. These findings may facilitate the development of novel food products or orally administered vaccines.

## Introduction

The domestic silkworm, *Bombyx mori*, is widely used for silk production. It is also used as a bioreactor in the mass production of recombinant bioactive proteins containing silk-based materials in the textile and pharmaceutical industries (1). Expressing proteins in silkworms via the baculovirus expression vector platform offers several advantages, including high expression levels, low costs, and the capacity to produce multiple proteins in large quantities, as well as the ability to perform co- and post-translational modifications characteristic of mammalian cells (2). This platform increases production and enhances the supply of various medium-scale recombinant proteins and has led to the rise of a promising vaccine production technology (3). In addition, it incorporates *piggyBac* transposon as well as genome-editing technologies to facilitate advanced utilization of transgenic silkworms (4–6). This platform has been used to produce genetically engineered strains that may generate biomedical (7) as well as recombinant silk materials (8–10).

Insects are known sources of nutrients and have been consumed traditionally to prevent and treat many human disorders (11–13). Edible insects, including silkworms, are a healthy and sustainable source of amino acids, fatty acids, and various micronutrients. Moreover, they contain bioactive compounds with antioxidant, antihypertensive, anti-inflammatory, antimicrobial, and immunomodulatory properties, which impart health benefits to humans (14–16). Genetically engineered silkworms have been used as a platform for producing food-grade oral vaccines that have been shown to induce strong immunoprotective responses at the preclinical level (17).

As a domestic species, silkworm larvae possess a huge silk gland, which synthesizes and secretes large amounts of protein. Moreover, this gland can store extremely high concentrations of liquid silk protein in soluble form sans aggregation or denaturation (18). The silk gland of a silkworm weighing approximately 2 g may produce up to 500 mg of silk proteins, accounting for approximately 25% of larval dry weight (19). Liquid silk is quickly converted into insoluble silk fibers just before being spun out to form a cocoon (20). Fibroin, the main component of silk proteins, accounts for 70% of the cocoon (21). It has many properties that are pertinent to biotechnology, such as biodegradability, biocompatibility, and robust mechanical strength characterized by high tensile strength (22). Silk proteins are also used in pharmaceuticals or as supplementary nutrients (23). Silk and fibroin hydrolysates lowered blood pressure and improved endothelial functions in spontaneously hypertensive rat models (12). In addition, they exhibited anti-diabetic properties, promoted skeletal muscle mass, reduced inflammation, and modulated gut microbiota in middle-aged female rats (24, 25). Furthermore, these hydrolysates inhibited the angiotensin-converting enzyme, which is strongly associated with hypertension and atherosclerosis (26).

Domestic silkworm larvae-based expression system is a promising platform that may be utilized for producing functional foods or oral vaccines. However, various bottlenecks exist to hamper development and utilization of such platforms, with particular reference to biosafety aspects linked to oral administration (17). Many effective strategies have been proposed to reduce the biological and allergenic risks stemming from edible insects. Physicochemical extraction methods, including the use of heat treatment or enzymatic hydrolysis, have been used to develop bioactive protein hydrolysates (27). However, many bioactive proteins may be susceptible to bio-physicochemical changes that occur during food/biomaterial processing. For example, heat treatment during the processing of food/biomaterial may modify the three-dimensional structure of proteins, leading to a loss of function in bioactive proteins as well as changes in immunochemical reactivity of vaccine antigens (14). Effective oral vaccines need to overcome harsh conditions, such as the presence of extremely low pH, proteolytic enzymes, and bile salts in the gastrointestinal environment. Furthermore, other factors, such as low permeability and immunogenicity of vaccines need to be addressed (28). Therefore, the preservation of protein bio-physicochemical and functional properties warrants further investigation to enhance bioavailability and reduce biological and allergenic risks associated with oral administration. However, reports pertaining to the nutritional and pharmaceutical mechanisms of silkworm materials remain scant, because of which many studies have resorted to investigating hydrolysates and heat-denatured silkworm materials. Furthermore, existing research is largely restricted to *in vitro* and animal studies. Therefore, the objective of this study was to investigate the inherent bio-physicochemical properties of edible silkworms to accurately evaluate their full potential, with an aim of facilitating the development of novel orally administrated food supplements or vaccines.

## Materials and methods

### Silkworm and mulberry leaves

The Kosetsu strain of *B. mori* was used in this study. Larvae were reared in the silkworm rearing room at the research farm of Shinshu University from spring to summer seasons, using mulberry leaves until just before spinning. The feces of larvae were collected just before spinning. Some larvae were fasted just before spinning and collected 2 days later to eliminate feces in the intestines. The mulberry leaves were collected from a mulberry garden in Shinshu University. Collected larvae and mulberry leaves were immediately washed with tap water and then frozen using liquid N2. They were then stored at −20°C until further use. Furthermore, silk glands of larvae were dissected in saline and stored at −20°C until further use.

### Preparation of silkworm larval powder

Stored frozen silkworms and other materials were lyophilized using a freeze dryer (FDM-2000; EYELA, Tokyo, Japan). A dry chamber (DRC-3 L; EYELA) was attached for 24 h at −80°C under 4 Pa vapor pressure. In some cases, the larvae were autoclaved for 60 min at 121°C prior to lyophilization. Then, 3 g of dried larvae were placed in a 50 mL collection tube (ST-5010PCR) containing a metal corn (MC-5038R; Yasui Kikai, Osaka, Japan). Dried larvae were pulverized using a MB3000 Multi-Beads Shocker (Yasui Kikai) at 3,000 rpm for 20 s. The percentage yields of resulting powders were determined by monitoring the weight of larvae and powder. Namely, the yield rate (%) was calculated as the percentage of weight of powder obtained from dry larva weight. Two types of silkworm powder were obtained; one was a raw condition powder that was not heat-treated, named “B100rw,” whereas the other was a powder that was denatured through autoclaving, named “B100dn” to compare the bio-physicochemical properties (29, 30).

### Appearance of powder

Surface images of B100rw were captured using optical microscopy (VHX8000; KEYENCE, Tokyo, Japan). The particle size distribution of B100rw and B100dn was measured using a laser particle size analyzer (MT-3300; NIKKISO, Tokyo, Japan). While the measurement time was 5 s, the refractive indexes were maintained at 1.81.

### Composition analysis

The composition of powders was analyzed via the STANDARD Tables OF FOOD COMPOSITION IN JAPAN-2015- (Seventh Revised Version) Analysis Manual,^1^ with some modifications. Briefly, protein content was measured via the combustion method using a DUMATHERM Npro (C. Gerhardt Japan, Tokyo, Japan) with a protein-to-nitrogen conversion factor of 6.25. Lipid content was measured using the acid hydrolysis method. The moisture content was determined via constant weight drying in an oven at 105°C. The ash content was determined via incineration in an electric furnace (FP-31, Yamato Scientific, Tokyo, Japan) at 550°C for 5 h. Carbohydrate content was calculated by subtracting the protein, fat, ash, and moisture content from 100 g of powder. Calories were calculated as the sum of the quantified contents of protein, fat, and carbohydrates multiplied by the Atwater factor (proteins = 4 kcal/g, fats = 9 kcal/g, carbohydrates = 4 kcal/g). Sodium, potassium, iron, and calcium contents were measured using an atomic absorption spectrophotometer (AA-6200; Shimadzu Corporation, Tokyo, Japan). The content of each α-tocopherol and β-carotene was determined by measuring the sample solution prepared using the direct saponification method through high-performance liquid chromatography (31).

### SDS-PAGE and proteomic analysis

B100rw and B100dn were dissolved by heating to 60°C in 9.3 M lithium bromide for 3 h, with gentle stirring. Then, the sample solution was dialyzed against water using a SnakeSkin dialysis membrane with a molecular weight cutoff of 3.5 kDa (Thermo Fisher Scientific, Waltham, MA, United States). The samples were mixed at a ratio of 1:1 (v/v) with a sample buffer [2% SDS, 125 mM Tris–HCl (pH 6.8), 5% 2-mercaptoethanol, 20% glycerol, and 0.02% Bromophenol Blue]. After mixing, all samples were electrophoresed on a 15% polyacrylamide gel. Furthermore, 1 mg of each powder was directly added to 250 μL sample buffer, mixed vigorously, and spun down. Next, 10 μL of the supernatant was electrophoresed. Precision Plus Protein Kaleidoscope Standards (Bio-Rad, Hercules, CA, United States) were used as protein standards. Electrophoresis was conducted at a current of 180 V for 90 min. Then, the gel was stained with CBB R-250 (FUJIFILM Wako, Osaka, Japan).

Before liquid chromatography–tandem mass spectrometry (LC–MS/MS) analysis, protein bands of interest on the SDS-PAGE gel were cut out. Then, the gels were treated with 10 mM dithiothreitol and alkylated with 55 mM iodoacetamide, followed by digestion with 12.5 mg/μL trypsin overnight at 37°C. Resultant peptides were separated via a 0–40% linear acetonitrile gradient for 30 min, followed by analyses with an LC–MS/MS (nanoACQUITY UPLC Xevo Qtof; Waters, MA, United States). Data were processed using ProteinLynx Global Server 3.0.3 and searched against *B. mori* and *Morus* L. protein entries in the UniProt Knowledgebase (UniProtKB).

### Trehalase activity

B100rw and B100dn were dissolved with extraction buffer [0.3 M NaCl, 5 mM EDTA, 10 mM HEPES-NaOH (pH 7.5)] of 10× volume, mixed vigorously for 5 min, and centrifuged at 20,000 × *g* for 30 min at 4°C. The supernatant, considered the soluble fraction, was directly used as the enzyme solution to determine the trehalase activity assay. A 20 μL enzyme solution was mixed with varying concentrations (0–10 mM) of a 230 μL trehalose solution in 0.1 M phosphate buffer saline (pH 6.0) and incubated at 37°C for 50 min. Phosphate (pH 3.0, 6.0–8.0, 11.0, and 12.0), acetic acid/sodium acetate (pH 4.0, 5.0), Tris-hydrochloric acid (pH 9.0), and carbonate–bicarbonate (pH 10.0) buffers were used to determine the optimal pH. Thereafter, the reaction was stopped by heating at 75°C for 10 min. Coagulated protein was removed via centrifugation at 20,000 × *g* for 5 min at 4°C, and an aliquot of the resulting supernatant (reaction solution) was used to measure the amount of glucose using the glucose oxidase-peroxidase (GOD-POD) method (32), with some modifications. The reaction was performed in a total volume of 1.1 mL containing 100 μL reaction solution and 1 mL GOD-POD solution [300 U of glucose oxidase (FUJIFILM Wako Pure Chemical Corp., Osaka, Japan), 0.5 mg of peroxidase (FUJIFILM Wako Pure Chemical Corp.), 1 mL of 20% (w/w) TritonX-100, 0.5 mg of DAB HCl (DOJINDO, Kumamoto, Japan) in 100 mL of 0.5 mM Tris-Cl (pH 7.0)] at 37°C for 50 min. To verify trehalase activity, a trehalase-specific inhibitor, validoxylamine A (VAA) (Toronto Research Chemicals Inc., Toronto, Canada), was added to the reaction mixture. Absorbance was measured at 465 nm. The amount of glucose catalyzed by trehalase was determined using the standard curve. Protein content was determined using a TAKARA BCA protein assay kit (Takara, Tokyo, Japan) with bovine serum albumin as the standard.

### Attenuated total reflectance/Fourier transform infrared spectral analysis

A Fourier Transform Infrared (FT-IR) device (IR Prestige-21, Shimadzu Corp.) equipped with an attenuated total reflectance (ATR) apparatus (DuraSamplIRII, Smiths Detection, London, England) was used to evaluate peaks derived from the protein secondary structures of all samples. For all measurements, each sample was collected with 60 accumulations at a 2.0 cm^−1^ resolution; the wavenumber ranged from 600 to 4,000 cm^−1^ (33, 34). Collected spectra were corrected against air as the background.

### Statistical analysis

Statistical parameters, including definitions and *n* values, are provided with relevant figures or corresponding figure legends. Statistical analyses were performed in Excel 2011 (Microsoft) using the software add-in Toukei-Kaiseki Ver. 3.0 (Esumi). Data are expressed as the mean ± standard deviation (SD). Statistical significance was set at *p < 0.05; **p < 0.01, ***p < 0.001; ns indicates no significant difference.

## Results and discussion

### Appearance of The silkworm larval powder

**Figure 1:**
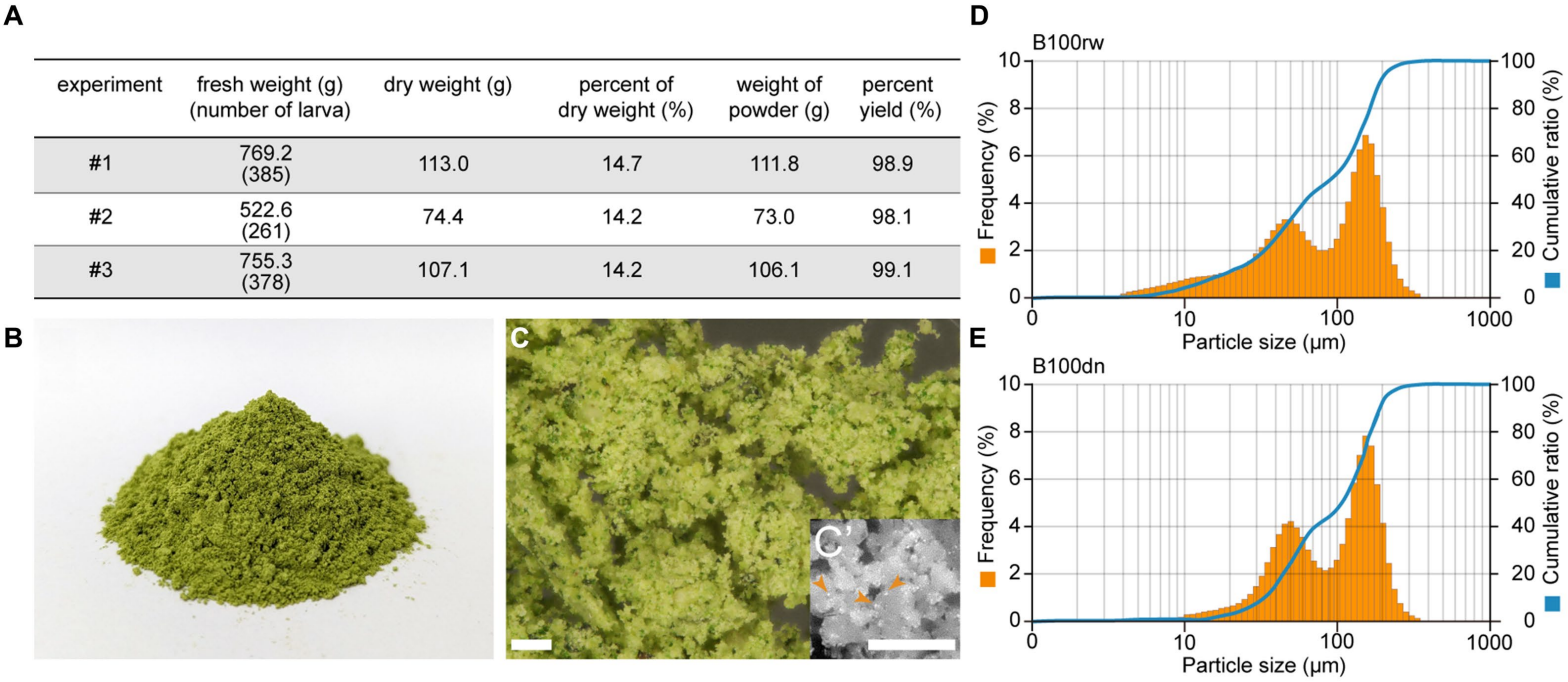
The appearance of silkworm larval powders, B100rw and B100dn. **(A)** The yield rate of B100rw. Percentage yield (%) was calculated using three independent experiments (#1–3). **(B)** Dark green appearance of B100rw. **(C)** Microscopic observation of B100rw. *Inset*: Enlarged view, bar = 200 μm. (**D**,**E**) Typical particle size distribution patterns in B100rw **(D)** and B100dn **(E)**. Orange bars and blue lines indicate the frequency (%) and cumulative ratio of particle size, respectively.

The powder was successfully prepared using the 5th instar larvae just before spinning, at a dry weight:fresh weight ratio of approximately 14%. Overall, the yield of the obtained powder was almost 100%, with 98.9, 98.1, and 99.1% yield obtained in three independent experiments (Figure 1A).

B100rw appeared dark green, which was probably attributable to mulberry leaves in the midgut content (Figure 1B). Microscopic observation revealed both greenish-brown and yellowish green particles, as well as fine highly bright white particles (<10 μm) in the B100rw powder (Figures 1B–C’). Although the source of the fine white particles is unknown, it may be liquid silk that makes up nearly 25% of the B100rw. Furthermore, the particle sizes of B100rw and B100dn were also measured (Figures 1D,E). The particle size distribution of each powder was represented by a bimodal peak consisting of a broader small first peak in the region of 47.98–52.33 μm and a defined second peak at 148.0–161.4 μm. However, B100dn distribution at <20 μm was drastically decreased and accordingly increased in frequency in its bimodal peak compared with that of B100rw (Figures 1D,E; Supplementary Figure S1). Autoclaving of food materials increases the water holding capacity and absorption rate, swelling capacity, bulk density, and particle size, consequently increasing the number of larger granules via adhesion between damaged granules (29, 30). This suggests that B100dn had been heat-denatured, and that we had successfully obtained two powders with different properties.

### Component analysis of powders

The nutritional components of each powder, the larva-derived B100rw, B100dn, fasted silkworm [B100rw(-Feces)], and feces as well as those of mulberry leaves and cocoon layer were quantified (Table 1). The midgut contents were removed from the fasted silkworm larvae to investigate the effects of mulberry as the diet. There was no significant difference between the nutritional component values of B100rw and B100dn, indicating that heat treatment had not affected basic nutritional composition. The protein, ash, and α-tocopherol composition of B100rw derived from silkworm larvae were similar to those reported by Rumpold and Schlüter (35), whereas the values observed for lipids, calcium, potassium, and β-carotene were higher. This variation is attributable to silkworm strain, diet, and rearing method, although high protein content was maintained (13).

**Table 1:** Composition of powders derived from silkworm larva and mulberry leaves.

Powder^1^	kcal^2^	Protein	Fat	Carbohydrate^3^	Moisture	Ash	Na	K	Fe	Ca	α-tocopherol	β-carotene
		(g)					(mg)				(mg)	
B100rw	406.3	58.7	11.9	16.1	3.0	10.3	7	3,378	4	578	5.6	2.5
B100dn	410.8	57.1	12.4	17.7	3.0	9.8	7	3,270	4	590	4.4	2.3
B100rw (-Feces)	407.6	71.4	11.6	4.4	3.0	9.6	11	3,127	3	235	1.8	0.5
Mulberry leaves	361.7	22.6	6.9	52.3	5.6	12.6	2	2,309	6	2,088	14.0	7.9
Feces	326.9	18.2	3.7	55.2	4.6	18.3	3	3,503	20	2,875	8.4	4.4
Cocoon layer	359.1	98.3	0.06	0.0	9.3	1.0	2	149	3	291	1.1	0.1

^1^All values are based on 100 g of powder. ^2^kcal = 4 × protein content + 9 × fat content + 4 × carbohydrate content. ^3^Carbohydrate content = 100 − (fat content + protein content + ash content + moisture content).

The potent hypoglycemic activity caused by 1-deoxynojirimycin (DNJ), which silkworms obtain by feeding on mulberry leaves, has been demonstrated (23). Therefore, we hypothesized that most of the carbohydrates, calcium, α-tocopherols, and β-carotene in B100rw and B100dn were also derived from mulberry leaves, which had accumulated in the intestines as feces. β-carotene exerts various effects, such as the regulation of biological functions, immune activation, and cell growth (36). However, cricket powder obtained from crickets fed a diet that does not include this nutrient, contains almost no β-carotene (35). Therefore, B100rw may be enriched, not only with DNJ, but also with other mulberry-derived components, which provide effects that are not obtainable with other insects, such as crickets. These findings suggest that B100rw is a sustainable source of silkworm and mulberry nutrients containing functional components that may positively impact human health.

### Proteomic analysis

**Figure 2:**
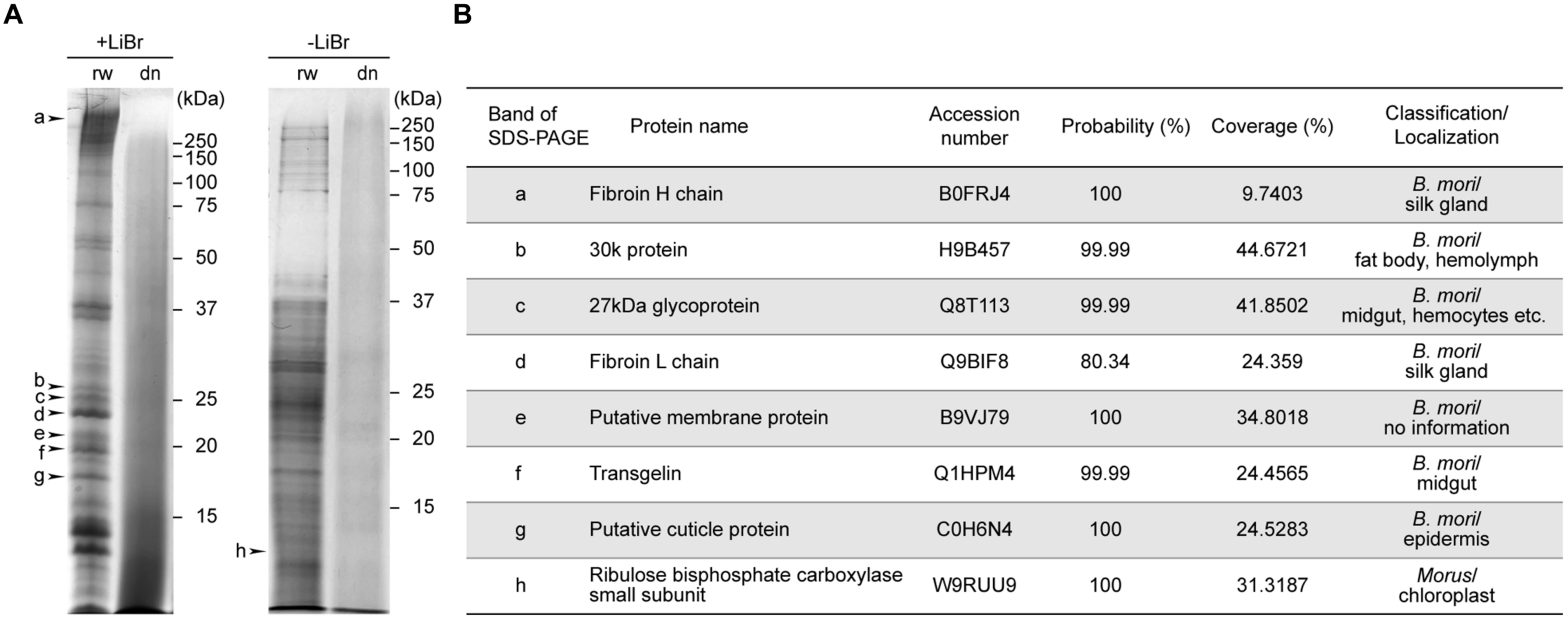
SDS-PAGE and LC–MS/MS analysis of B100rw and B100dn. **(A)** SDS-PAGE analysis of protein components in B100rw and B100dn. +LiBr and − LiBr indicate samples prepared by adding or excluding lithium bromide, respectively, during protein solubilization. The bands indicated by arrowheads were cut off and subjected to LC–MS/MS analysis. **(B)** A list of identified proteins corresponding to peptides obtained through a database search, which describes the probability (%) and coverage (%) in LC–MS/MS analysis.

Each protein subunit contained in B100rw and B100dn were compared using SDS-PAGE (Figure 2A). We performed two types of sample preparation. LiBr was added to the powders to solubilize insoluble proteins, such as a fibroin (Figure 2A, +LiBr). Many bands were detected with B100rw, but a smear was detected with B100dn both with and without LiBr elution (Figure 2A). This suggested that autoclaving had degraded B100dn protein (37). We then cut each band from SDS-PAGE gels (Figure 2A; band a–h), performed in-gel trypsin digestion, and analyzed products via LC–MS/MS, followed by a database search (Figure 2B). Based on this result, proteins with a probability (%) exceeding 80% and the highest coverage (%) were used for identification. We identified the various peptide sequences of the B100rw sample, which contained the fibroin complex, consisting of a fibroin heavy chain (Fib-H, approximately 350 kDa) (Figure 2A; band a), fibroin light chain (Fib-L, approximately 26 kDa) (Figure 2A; band d), and P25/fibrohexamerin (fhx/P25) (38). In addition, the identified subunits were mainly derived from a 30 K protein (39), 27 kDa glycoprotein (40), putative membrane protein, transgelin (41), putative cuticle protein (42), and ribulose bisphosphate carboxylase (43). These were likely derived from the silk gland, fat body, hemolymph, midgut, hemocytes, epidermis, and mulberry leaves (Figure 2B). These findings indicated that B100rw contained non-degraded larval proteins and mulberry leaf-derived proteins.

### Monitoring trehalase activity

**Figure 3:**
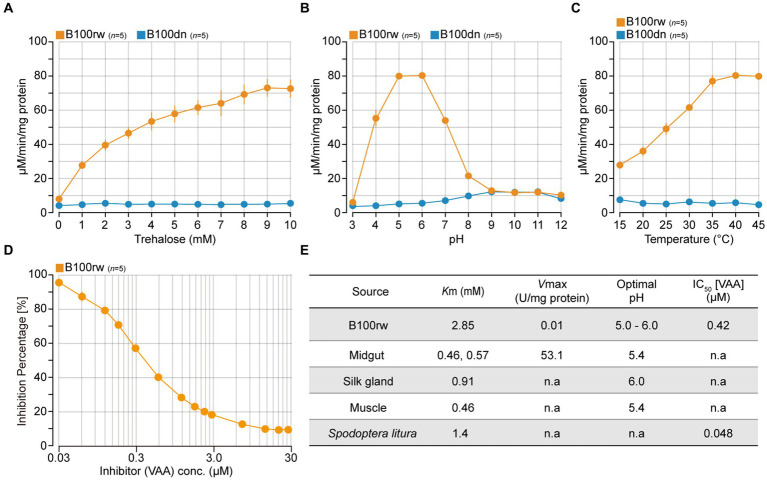
The activity properties of soluble trehalase extracted from B100rw and B100dn. Enzyme activities were measured using soluble fractions extracted from B100rw (orange) and B100dn (blue). **(A)** Substrate (trehalose) concentration and activity relationship. **(B)** Determination of optimal pH. **(C)** Effects of temperature, as examined at 15–45°C. **(D)** Effects of validoxylamine A, as examined at 0.03–30 μM. **(E)** Comparison of physical and kinetic properties of trehalase between the B100rw extract and soluble trehalase in various tissues. The Michaelis constant (*K*_m_) and maximum rate of the reaction (*V*_max_) were calculated using Hanes-Woolf plots. The values of *K*_m_, *V*_max_, and optimal pH of soluble trehalase in the midgut (44, 45), silk gland (46), and muscle (45). n.a indicates no analysis.

We measured trehalase activity to determine whether B100rw maintained enzymatic activity (Figure 3; Supplementary Figure S2). Insect trehalase is a glycosidase that catalyzes the irreversible breakdown of trehalose, a major hemolymph sugar, which acts as an instant source of energy (47, 48). This sugar is broken into two glucose units and forms a link between trehalose metabolism and glycolysis, including in *B. mori* (49–51). In silkworms, trehalase is ubiquitously localized to various tissues, including the midgut, muscle, silk gland, and cocoon layer (44–46, 52). Additionally, the amino acid sequences of trehalase indicate stability at high temperatures (53, 54). Therefore, we selected trehalase as an indicator enzyme to monitor biochemical activity in B100rw. This enzyme exists in two distinct forms, which are Tre-1, the soluble form and Tre-2, the membrane-bound form found in *B. mori* (47, 55). As we prepared the enzyme solution-derived soluble fraction of B100rw, we assumed that Tre-1 activity was monitored during this study.

We examined the relationship among substrate concentration, pH, temperature, and an inhibitor in trehalase activities of the soluble fraction of B100rw or B100dn extracts (Figures 3A–D). The dependency of the catalytic activity of B100rw extract on substrate concentration exhibited a reaction curve that increased steadily up to 8–9 mM and reached a plateau at 10 mM of trehalose (Figure 3A). From the Hanes-Woolf plots, the value of *K*_m_ was calculated to be 2.85 mM and that of *V*_max_ to be 0.01 U/mg protein (Figure 3E; Supplementary Figure S2A). The *K*_m_ value was high, and *V*_max_ was considerably high compared with previously reported kinetics parameters (44–46). This suggests that the part of trehalase may be degraded and/or that soluble trehalase in the soluble fraction could not be extracted.

The activity-pH relationship of the B100rw extract showed a typical bell-shaped curve with a single peak at pH 5.0–6.0, similar to that of previous reports (Figure 3B). Furthermore, the effect of temperature on the rate of B100rw extract activity was examined at 15–45°C (Figure 3C). B100rw activity increased steadily until 35°C and then reached a plateau. The effect of VAA, a trehalase-specific inhibitor, was evaluated. VAA inhibited trehalase activity of the B100rw extract at varying concentrations in a competitive manner, the half-maximal inhibitory concentration (IC_50_) value being 0.42 μM (Figures 3D,E; Supplementary Figure S2B). This value was high, compared to that of trehalase extracted from *Spodoptera litura* larvae (56). By contrast, no trehalase activity was detected in the soluble fraction of the B100dn extract (Figures 3A–C). These results demonstrated that trehalase activity was maintained in the B100rw extract, owing to its retained conformation.

### Protein secondary structural analysis using FT-IR spectroscopy

Hu et al. (33) identified spectral parameters related to protein secondary structures in the amide-I region (*ca.* 1,680–1,580 cm^−1^). The protein secondary structures, namely, the α-helix/random coil (*ca.* 1,645 cm^−1^) and β-sheet (*ca.* 1,620 cm^−1^), were reflected in the peak position within this region. Therefore, protein secondary structures were compared based on their relative peak positions.

**Figure 4:**
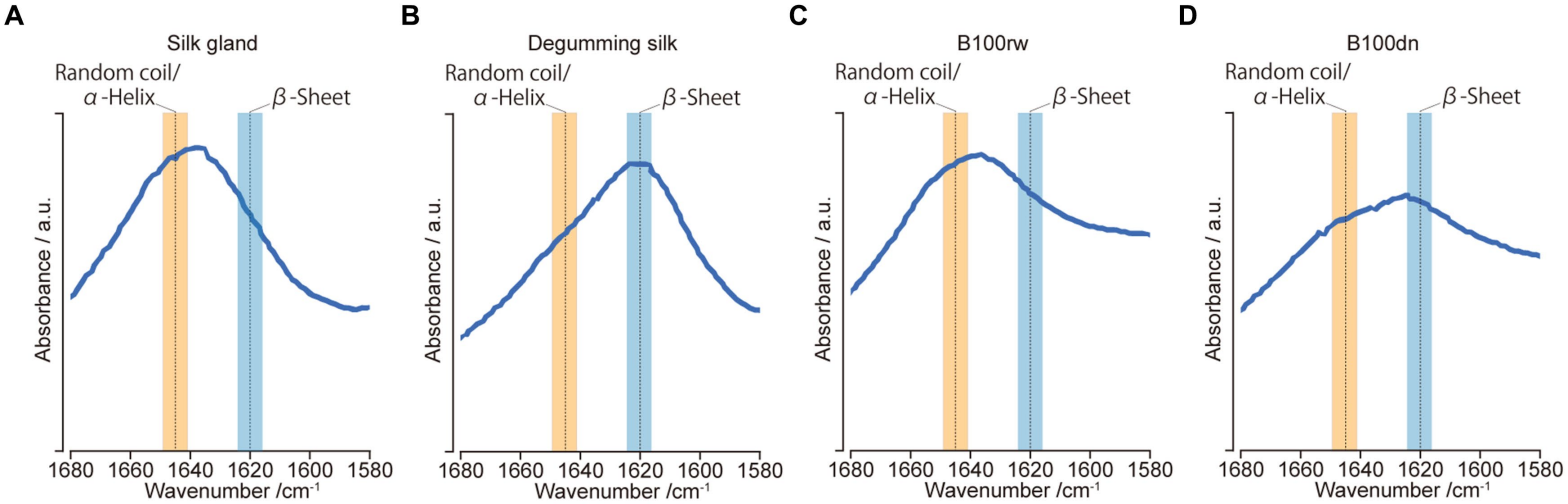
FT-IR analysis. FT-IR spectra of amide-I regions between 1,680 and 1,580 cm^−1^ for samples derived from silk glands **(A)**, degumming silk **(B)**, B100rw **(C)**, and B100dn **(D)**. Orange and blue bands represent the ranges of random coils/α-helices and β-sheets, respectively.

FT-IR spectroscopy was used to investigate the protein secondary structures in B100rw and B100dn, as well as in silk gland and degumming silk powders (Figure 4). First, we confirmed the FT-IR spectra in the amide-I region in the silk gland and degumming silk. The structures of liquid silk proteins are aligned via spinning and β-sheet increases (57, 58). In the present study, amide-I peaks were observed at 1,636 and 1,617 cm^−1^, indicating the presence of random coils, α-helix structures, and a β-sheet, respectively (Figures 4A,B). In B100rw and B100dn, the amide-I bands at 1,636 and 1,624 cm^−1^ represented random coils/α-helix and β-sheets, respectively (Figures 4C,D). Thus, the FT-IR spectrum of B100dn was altered compared to that of B100rw.

Thermal denaturation leads to the rearrangement of protein structures into a more stable energy state, such as β-sheet (59, 60). This suggested that the differences in the B100rw and B100dn peaks may reflect the effects of autoclaving. Furthermore, the peak of B100rw was close to that of the silk gland. Thus, we speculate that the silk protein in B100rw may have been predominantly powdered while maintaining a liquid crystalline structure. This hypothesis was supported by the fact that silk glands account for nearly 25% of the weight of B100rw. Therefore, the structure of silk protein could possibly influence the physical and functional properties of B100rw. However, the effects exerted by the preferential random coils/α-helix structure of B100rw on oral administration remains unclear, indicating the need for further research.

In conclusion, the present study characterized raw silkworm larval powder, B100rw. This powder was prepared from whole larvae and contained non-denatured protein. Thus, it may retain nutritional or pharmaceutical effects that are lost during processing. Studies have shown that the larval powder of silkworm is a healthy and sustainable source of bioactive compounds that can impart health benefits to humans (23). Therefore, B100rw extract may represent the inherent biomedical properties of silkworms and thereby potentially benefit humans. Ingestion of silkworm may provide liquid silk, which mainly contains fibroin, with unique properties that are particularly advantageous as useful food and biomedical materials. In addition, genetic engineering techniques may be utilized to enhance nutritional and pharmaceutical value of silkworm-based products. However, silkworms contain allergens in each developmental stage and in their metabolites. Forty-five potential allergens have been reported in the developmental stages of silkworms, as well as in their silk and feces so far (23, 61). Thus, our findings indicate that novel orally administered food or vaccines may be developed by refining B100rw extract via innovative processing technologies that help retain and enhance its nutritive value and functionality, thereby paving the way for innovative insect-based food products.

## Data availability statement

The raw data supporting the conclusions of this article will be made available by the authors, without undue reservation.

## Ethics statement

The manuscript presents research on animals that do not require ethical approval for their study.

## Author contributions

SH: Visualization, Methodology, Investigation, Formal analysis, Data curation, Writing – original draft. MY: Writing – original draft, Visualization, Methodology, Investigation, Formal analysis, Data curation. HU: Visualization, Methodology, Investigation, Formal analysis, Data curation, Writing – original draft. SY: Writing – review & editing, Investigation, Formal analysis, Data curation. MK: Writing – review & editing, Investigation. TY: Writing – review & editing, Resources. KY: Writing – review & editing, Validation, Supervision, Formal analysis. KS: Writing – review & editing, Visualization, Validation, Supervision, Project administration, Investigation, Funding acquisition, Formal analysis, Data curation, Conceptualization, Writing – original draft.

## Footnotes

^1^ https://www.mext.go.jp/a_menu/syokuhinseibun/1368931.htm

## Supplementary material

SUPPLEMENTARY FIGURE S1 Particle size distribution of B100rw and B100dn in the cumulative frequency at 20 µm intervals. Each bar represents the mean ± SD of three samples; *, P<0.05; ***, P<0.001.

SUPPLEMENTARY FIGURE S2 Calculation of kinetic properties of trehalase extracted from B100rw. **(A)** Hanes-Woolf plots for trehalose concentration and activity relationship. *V* and *S* indicate the relative specific activity of trehalase and trehalose concentration, respectively. A linear function (*y* = 100.83*x* + 286.95) was obtained. The slope is 1/*V*max, and the *X*-intercept is −*Km*. **(B)** Inhibition by validoxylamine A (VAA). Relative activities were determined at various inhibitor concentrations, with no inhibitor represented as 100%. The function was created using the five central points, and the function was *y* = −22.62ln(*x*) + 5.5994. IC_50_ was calculated at 0.42 µM.
