# Supplementary material for: Evaluating bio-physicochemical properties of raw powder prepared from whole larvae containing liquid silk of the domestic silkworm
Source: Front Nutr. 2024 Jun 5;11:1404489. doi: 10.3389/fnut.2024.1404489 (PMC11188413; doi:10.3389/fnut.2024.1404489)
Supplement: SUPPLEMENTARY FIGURE S1 — Particle size distribution of B100rw and B100dn in the cumulative frequency at 20 µm intervals. Each bar represents the mean ± SD of three samples; *, P<0.05; ***, P<0.001. [file Image_1.pdf]

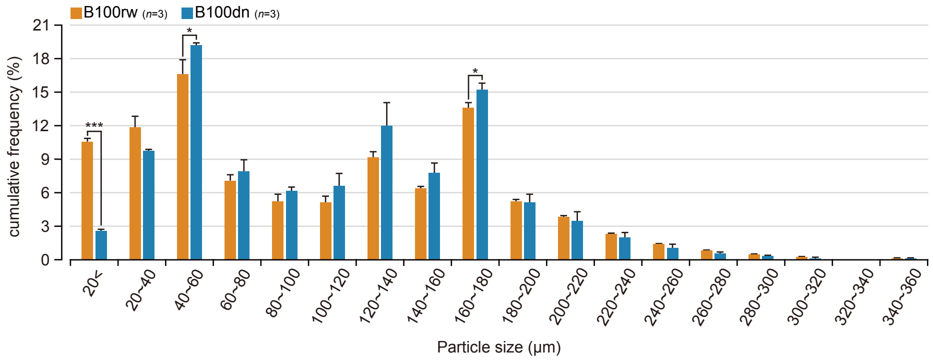

Supplementary Figure 1. Particle size distribution of B100rw and B100dn in the cumulative frequency at 20 μm intervals. Each bar represents the mean ± SD of three samples; *, $P < 0.05$; ***, $P < 0.001$.
