# Supplementary material for: Evaluating bio-physicochemical properties of raw powder prepared from whole larvae containing liquid silk of the domestic silkworm
Source: Front Nutr. 2024 Jun 5;11:1404489. doi: 10.3389/fnut.2024.1404489 (PMC11188413; doi:10.3389/fnut.2024.1404489)
Supplement: SUPPLEMENTARY FIGURE S2 — Calculation of kinetic properties of trehalase extracted from B100rw. (A) Hanes-Woolf plots for trehalose concentration and activity relationship. V and S indicate the relative specific activity of trehalase and trehalose concentration, respectively. A linear function (y = 100.83x + 286.95) was obtained. The slope is 1/Vmax, and the X-intercept is −Km. (B) Inhibition by validoxylamine A (VAA). Relative activities were determined at various inhibitor concentrations, with no inhibitor represented as 100%. The function was created using the five central points, and the function was y = −22.62ln(x) + 5.5994. IC50 was calculated at 0.42 µM. [file Image_2.pdf]

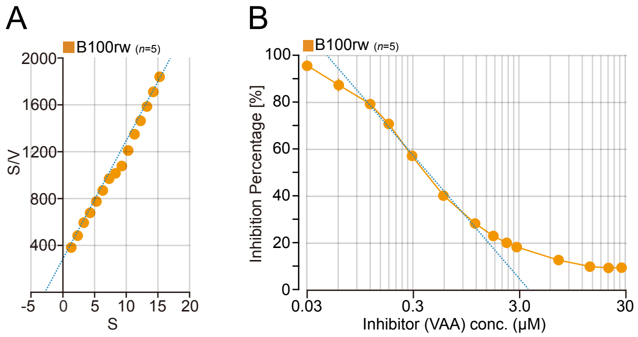

Supplementary Figure 2. Calculation of kinetic properties of trehalase extracted from B100rw. (**A**) Hanes-Woolf plots for trehalose concentration and activity relationship. V and S indicate the relative specific activity of trehalase and trehalose concentration, respectively. A linear function ($y = 100.83x + 286.95$) was obtained. The slope is $1/V$max, and the X-intercept is $-K$m. (**B**) Inhibition by validoxylamine A (VAA). Relative activities were determined at various inhibitor concentrations, with no inhibitor represented as 100%. The function was created using the five central points, and the function was $y = -22.62\ln(x) + 5.5994$. IC_{50} was calculated at 0.42 µM.
